# Coffee Husk By-Product as Novel Ingredients for Cascara Kombucha Production

**DOI:** 10.4014/jmb.2310.10004

**Published:** 2023-12-08

**Authors:** Bao Xuyen Nguyen Le, Thach Phan Van, Quang Khai Phan, Gia Bao Pham, Hoa Pham Quang, Anh Duy Do

**Affiliations:** 1 Department of Life Science, College of Natural Sciences, Hanyang University, Seoul 04763, Republic of Korea; 2 Department of Biotechnology, NTT Hi-Tech Institute, Nguyen Tat Thanh University, Ho Chi Minh City 700000, Vietnam

**Keywords:** Cascara, kombucha, fermentation, antioxidant, antibacterial, sustainable development

## Abstract

Kombucha, a fermented beverage, is gaining popularity due to its numerous beneficial health effects. Various substrates such as herbs, fruits, flowers, and vegetables, have been used for kombucha fermentation in order to enhance the flavor, aroma, and nutritional composition. This study aims to investigate the potential suitability of cascara as a novel ingredient for kombucha production. Our findings suggested that cascara is a suitable substrate for kombucha production. Fermentation elevated the total phenolic and flavonoid content in cascara, which enhanced the antioxidant, antibacterial, and prebiotic characteristics of the product. Furthermore, the accumulation of acetic acid-induced the pH lowering reached 2.7 after 14 days of fermentation, which achieved the microbiological safety of the product. Moreover, 14 days of fermentation resulted in a balanced amalgamation of acidity, sweetness, and fragrance according to sensory evaluation. Our findings not only highlight the potential of cascara kombucha as a novel substrate for kombucha production but also contribute to repurposing coffee by-products, promoting environmentally friendly and sustainable agricultural development.

## Introduction

The coffee processing industry generates a substantial volume of waste and by-products, primarily husks, accounting for approximately 45-50% of the total coffee harvest [1]. Vietnam is the second-largest coffee producer and exporter globally, with a cultivation area of over 695,000 hectares and an annual production of 1.76 million tons [2], accompanied by 800,000 tons of coffee husks annually. Lacking efficacy in agriculture waste management might negatively affect the ecosystem [1].

Repurposing of agricultural by-products is becoming increasingly crucial in promoting sustainable agricultural progress and minimizing adverse environmental effects [3]. In recent years, there has been an increasing emphasis on the commercial use of coffee husks, commonly known as cascara, despite their prior classification as coffee industry waste [4]. Cascara exhibits a notable abundance of antioxidants, fiber, and bioactive substances, hence placing it as a potentially beneficial beverage for the promotion of health [4]. Commercialization of cascara presents a dual advantage in the coffee business, as it not only mitigates the issue of by-product waste but also yields valuable goods that hold considerable potential for enhancing human health.

Kombucha is a fermented beverage that originated in China, which is produced by fermenting sweet tea with a symbiotic culture of bacteria and yeast (SCOBY) [5]. During fermentation, the sugar and tea are converted into organic acids and carbon dioxide, resulting in a beverage with a slightly fizzy and tangy taste [5, 6]. Kombucha also contains beneficial bacteria such as *Lactobacillus* spp., *Bacillus* spp., and *Bacteroides* species, would help maintain a healthy gut microbiome [7]. In contrast, the inherent acidity of kombucha contributes to the decrease of harmful bacteria in the intestinal environment [8]. Throughout the fermentation process, bacteria within the SCOBY catalyze the transformation of complex polyphenols into biologically active forms such as catechins, tannins, and phenolic acids. These compounds have been associated with various health benefits, including their roles as antioxidants and anti-inflammatory agents [9]. Furthermore, the process of kombucha fermentation facilitates the synthesis of flavonoids, which play a pivotal role in the potential health advantages [10]. The ingestion of kombucha offers a naturally occurring supply of polyphenols and flavonoids, which have been linked to several health advantages, including immune system regulation, cardiovascular well-being, and anti-cancer activity [10].

Kombucha is conventionally produced by fermenting black tea and sugar [11]. Several plant-derived substances, such as fruits, vegetables, herbs, spices, and flowers, have been the subject of research as prospective alternatives to black tea [12, 13]. This innovative approach opens avenues for diversifying kombucha flavors, catering to diverse taste preferences. Cascara, which refers to the dried husk of the coffee cherry, is a readily available residual material that contains a significant amount of bioactive phytochemicals such as chlorogenic acid, flavonoids, caffeine, and dietary fiber [14]. Cascara has various desirable qualities including antioxidant, anti-inflammatory, antibacterial, and lipolytic activities, making it a potentially valuable substrate for the development of kombucha products. The aim of this study was to develop an innovative kombucha beverage using cascara ingredients and afterward evaluate its biological characteristics. This endeavor represents a commercially viable value-added application for the cascara by-product, while also providing environmental benefits through enhanced sustainable agriculture practices and reduced coffee industry waste.

## Material and Methods

### Cascara and SCOBY

The cascara used in this study was sourced from Arabica coffee husks, which were purchased from a coffee plantation located in Dak Lak, Vietnam. The kombucha starter cultures SCOBY were procured from Foodplus Ltd in Ha Noi, Viet Nam. The microbial composition of SCOBY includes yeast *Brettanomyces bruxellensis*, *Saccharomyces cerevisiae*, acetic acid bacteria (AAB) including *Komagataeibacter pomaceri*, and *Komagataeibacter rhaeticus* at a density approximately of 5 × 10^6^ CFU/g, has been verified by VNU - Institute of Microbiology and Biotechnology, Vietnam National University, Hanoi, Vietnam. The starter culture was stored at 4°C and consisted of a sour broth and SCOBY.

### Bacterial Strains

Various pathogenic and probiotic bacterial strains were utilized in this study. The pathogenic including *Salmonella enterica* ATCC 14028, *Escherichia coli* ATCC 25922, and *Staphylococcus aureus* MRSA ATCC 43300, were purchased from the American Type Culture Collection (ATCC), while Vibrio cholerae was isolated from seafood. The probiotic bacteria including *Lactobacillus rhamnosus* ATCC 9595 acquired from the ATCC, *L. brevis* derived from fermented black tea kombucha, *Weizmannia coagulans* isolated from fermented dairy, and *Lactococcus lactis* obtained from fermented cabbage. The bacterial strains were provided by the Microbiology Laboratory culture collection at the Department of Biotechnology, NTT High Technology Institute, Nguyen Tat Thanh University.

### Kombucha Fermentation

The tea extracts used in this study were prepared by steeping 10 g/l of dry cascara, black tea, or oolong tea in hot water for 20 min. After steeping, aliquots of 90 ml of the extracts were transferred into 100 ml bottles. Varying amounts of sucrose were introduced into the bottles to accomplish final concentrations of 0, 50, 100, and 200 g/l. The supplemented extracts were then cooled down to ambient temperature. Next, SCOBYs were introduced into each bottle at a dose of 30 g/l. An additional 10% of previously fermented sour broth was also added along with the SCOBY. The cascara extract bottles were then incubated at 30°C. Measurements of the SCOBYs mass were conducted on the 7th, 14th and 21st day of incubation to monitor the progress of fermentation.

### Examination of pH and Acetic Acid Content in Cascara Kombucha

The pH of the cascara kombucha samples was measured at 0, 7, 14, and 21 days of fermentation using a Mettler Toledo J12683 pH meter (USA). Total titratable acidity was analyzed by performing an acid-base titration assay, using 0.1N sodium hydroxide (NaOH) solution. Phenolphthalein was utilized as the colorimetric indicator to detect the endpoint of the titration. The total acidity values obtained were expressed as acetic acid concentration (mg/l) of the fermented cascara kombucha [15].

### Quantification of Total Polyphenols Content (TPC) in Kombucha

The determination of total polyphenol content (TPC) in the kombucha samples followed the ISO 14502-1 standard protocol, employing the Folin-Ciocalteu colorimetric method originally outlined by Singleton and Rossi [16]. For this assay, 500 μl of the kombucha sample was dissolved with an equal volume of deionized water and 10%Folin-Ciocalteu reagent (500 μl) to form a reaction mixture. Subsequently, 500 μl of 10% sodium carbonate (Na_2_CO_3_) solution was introduced, and the mixture was underwent incubated at 40°C for 30 min. Spectrophotometric measurement of the absorbance at a wavelength of 765 nm was performed. TPC quantification was achieved by extrapolation from a calibration curve prepared using gallic acid within the concentration range of 0 to 100 μg/ml.

### Quantification of Total Flavonoids Content (TFC) in Kombucha

The quantification of total flavonoid content (TFC) followed the procedure outlined by Pękal A and Pyrzynska [17]. An analytical standard curve for quercetin (QE) was constructed with concentrations ranging from 25 to 400 μm/ml. The assay involved combining 0.5 ml of the kombucha sample or QE standard solution (diluted in 1.5 ml of ethanol) and allowing it to equilibrate for 5 min. Subsequently, 0.1 ml of 10% aluminum chloride (AlCl_3_) was added, followed by another 5-min incubation. Next, 0.1 ml of 1M potassium acetate (CH_3_COOK) and 2.8 mL of deionized water were introduced to the reaction mixture, ensuring thorough homogenization. The mixture was then incubated at room temperature for 45 min. Finally, the absorbance of both the experimental and standard mixtures was measured at 415 nm. The determination of TFC in the kombucha samples was accomplished by establishing a correlation between the measured absorbance values and the QE calibration curve.

### Investigation of Free Radical Scavenging of Kombucha Using DPPH Assay

The antioxidant activity of the kombucha samples was evaluated through a spectrophotometric assay using the stable free radical 2,2-diphenyl-1-picrylhydrazyl (DPPH) [18]. A 40 mg/l DPPH (TCI, Cas 1898-66-4, Japan) working solution was prepared. The reaction mixture was constituted by thoroughly mixing 1 ml of DPPH reagent with 50 μl of kombucha sample, followed by incubation in the dark at room temperature for 60 min. Post-incubation, the residual DPPH radical absorbance was recorded spectrophotometrically at 517 nm wavelength. The free radical scavenging capacity was computed using the following formula:

% inhibition = [(A0 − As) /A0] × 100

where:

A0: absorbance of the DPPH solution at 518 nm without the tested sample

As: absorbance of the DPPH solution at 518 nm with the tested sample.

### Investigation of free radical scavenging of kombucha using ABTS assay

The ABTS+ [2,2'-azino-bis(3-ethylbenzothiazoline-6-sulfonic acid)] radical cation decolorization assay was performed as described by Cano [19]. ABTS+ was generated by reacting 7 mM ABTS (Cool Chemical Science and Technology, China) and 2.45 mM potassium persulfate (K2S2O8, Xilong, China) at a 1:1 (v/v) ratio in phosphate-buffered saline (PBS) and incubating in the dark for 12-16 h at 4°C. The assay was carried out by combining 50 μl of kombucha sample with 1 ml of ABTS+ solution and incubating in the dark for 30 min at 25°C, followed by absorbance measurement at 734 nm. The free radical scavenging ability was determined using the following formula:

% inhibition = [(A0 − As) /A0] × 100

where:

A0: absorbance of the ABTS solution at 734 nm without the tested sample

As: absorbance of the ABTS solution at 734 nm with the tested sample.

### Investigation of Antibacterial Activity of Kombucha

The antibacterial efficacy of the kombucha preparations was investigated using the agar well diffusion method, following the protocol outlined by Balouiri [20]. The pathogenic bacteria were incubated in Mueller Hinton broth (MHB) (India) at a temperature of 37°C overnight. Subsequently, these cultures were diluted to an OD_600_ of 0.1, equivalent to a concentration of 1 × 10^7^ CFU/ml. One hundred μl aliquots of these bacterial suspensions were evenly spread onto Mueller Hinton agar (MHA) plates. Then, three wells, each with a diameter of 5 mm, were carefully created in the agar, evenly spaced from one another. To each of these wells, 100 μl of kombucha extract was added, while 100 μl of sterile distilled water was used as a negative control. The plates were then incubated at 37°C for a period of 24 h, during which the inhibition zones around each well were observed and measured.

### Determination of Prebiotic Activity of Cascara Kombucha

The probiotic strains were individually cultivated at a concentration of 1 × 10^6^ CFU/ml in glucose-free De Man, Rogosa and Sharpe (MRS) medium (Himedia, India), using 20% cascara kombucha as the carbon source instead of glucose. Positive control cultures were grown in standard MRS broth containing 2.0% (w/v) glucose, while negative controls were incubated in glucose-free MRS. After 48 h of incubation at 37°C, the growth of each probiotic isolate was quantified by serial decimal dilutions in PBS, followed by spread plating onto MRS agar and enumerating the resultant colony-forming units (CFU/ml) [21].

### Sensory Evaluation of Cascara Kombucha

A group of 20 expert evaluators employed a 9-point hedonic scale, ranging from 1 (strong dislike) to 9 (strong liking), to perform sensory analysis on the cascara kombucha preparations [22]. The samples evaluated included cascara kombucha fermented for 7, 14, and 21 days, with three independent batches per time point. The sensory attributes analyzed comprised appearance, color, sourness, sweetness, aroma, and astringency.

### Statistical Analysis

The study employed a completely randomized design (CRD) with three replicates for each treatment. The data were subjected to analysis using SAS 9.4 software (SAS, Inc., USA) and then reported as the mean ± standard error of the mean, derived from triplicate readings. The statistical significance between groups was assessed using Duncan's test, where a *p*-value < 0.05 was considered to indicate significance.

## Results

### Influence of Sucrose Concentration on the Development of Nascent Pellicles in SCOBYs Cultured in Cascara Substrate across Various Time Intervals

**Fig. 1:**
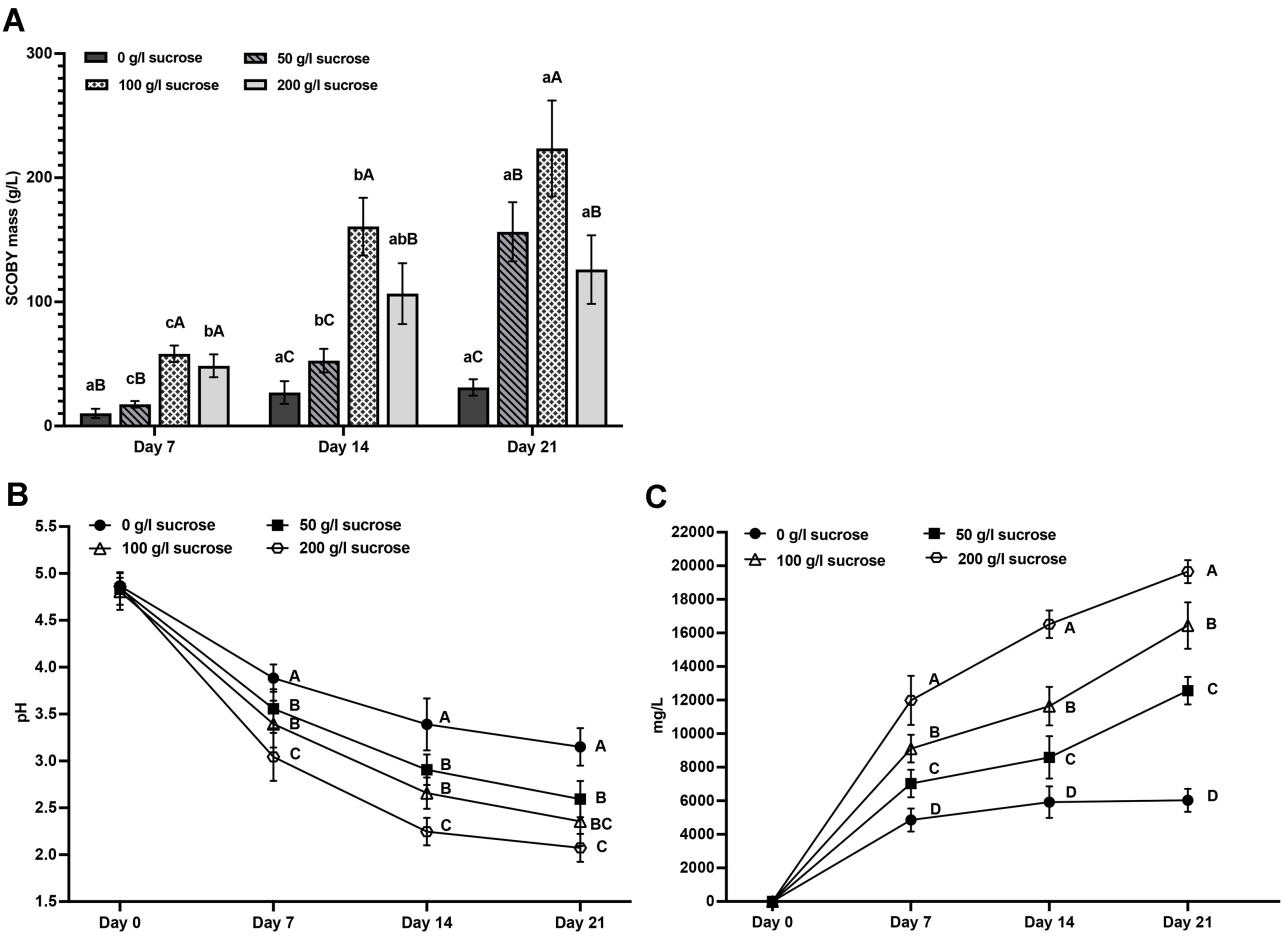
Effects of sucrose concentration on the growth of nascent pellicles SCOBYs (A) pH values (B) and acetic acid content (C) in cascara kombucha at different time intervals. Data are presented as the means of triplicate analysis ± standard deviation. Lowercase letters (a-c) indicate statistically significant differences between groups sharing the same sugar concentration (corresponding to columns of the same color) according to fermentation time interval (*p* < 0.05). The superscript letters (**A-C**) indicate significant differences between groups with sucrose concentrations of 0, 50, 100, and 200 g/l, respectively, at each fermentation time point (*p* < 0.05).

The biomass accumulation of nascent SCOBY pellicles cultivated on cascara medium supplemented with varying sucrose levels was monitored at 7, 14, and 21 days (Fig. 1A). A substantial increase in SCOBY weight was observed within 7 days, indicating rapid microbial proliferation, which further doubled by 21 days of incubation. Elevated sucrose concentrations correlated with a greater decline in pH and enhanced acetic acid production over fermentation time (Fig. 1B and 1C). Maximal SCOBY expansion was promoted at 100 g/l sucrose, while 200 g/l sucrose exhibited an inhibitory effect on growth (Fig. 1A). Thus, an optimal sucrose level of 100 g/l was selected for further investigation.

### Quantification of Total Polyphenols (TPC) and Flavonoids (TFC) Contents in Cascara Kombucha during Fermentation

**Fig. 2:**
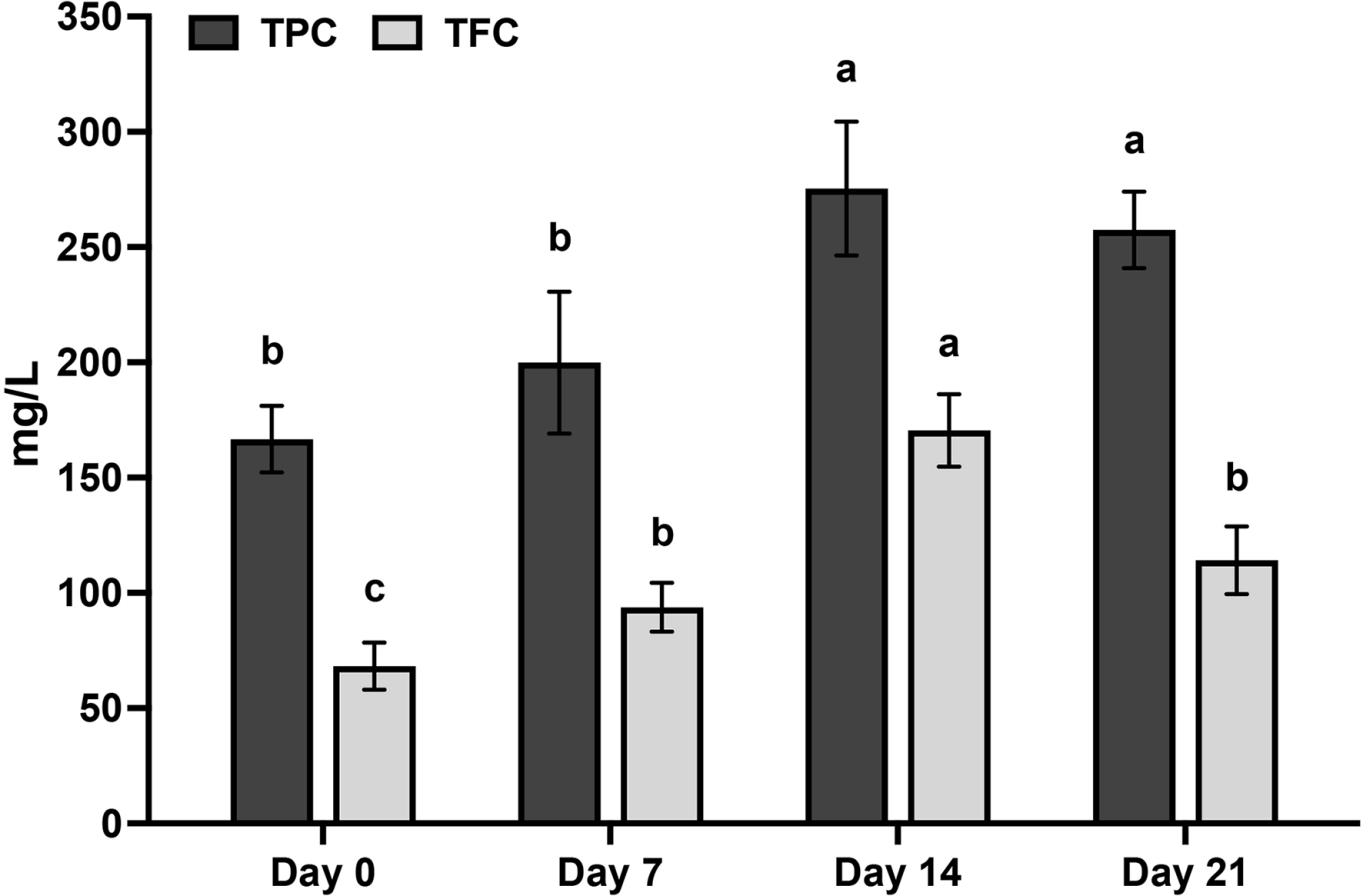
The total polyphenols content (TPC) / flavonoids content (TFC) in cascara kombucha fermented at sucrose concentration of 100 g/l at different time intervals. Data are presented as the means of triplicate analysis ± standard deviation. Lowercase letters (a-c) indicate significant differences between groups over time (*p* < 0.05).

The quantification of the total polyphenol content (TPC) and total flavonoid content (TFC) of cascara kombucha was conducted at various fermentation intervals. The initial TPC of the cascara extract was determined to be 166.69 mg/l, and substantial increase during the process of bioconversion, reaching its peak value of 275.41 mg/l after 14 days (Fig. 2). The increase in TFC was observed in the same pattern, reaching 170.44 mg/l after 14 days of fermentation (Fig. 2). However, both TPC and TFC subsequently exhibited a decline by the 21st day, as shown in Fig. 2.

### Investigation of Antioxidant Activities of Cascara Kombucha

**Fig. 3:**
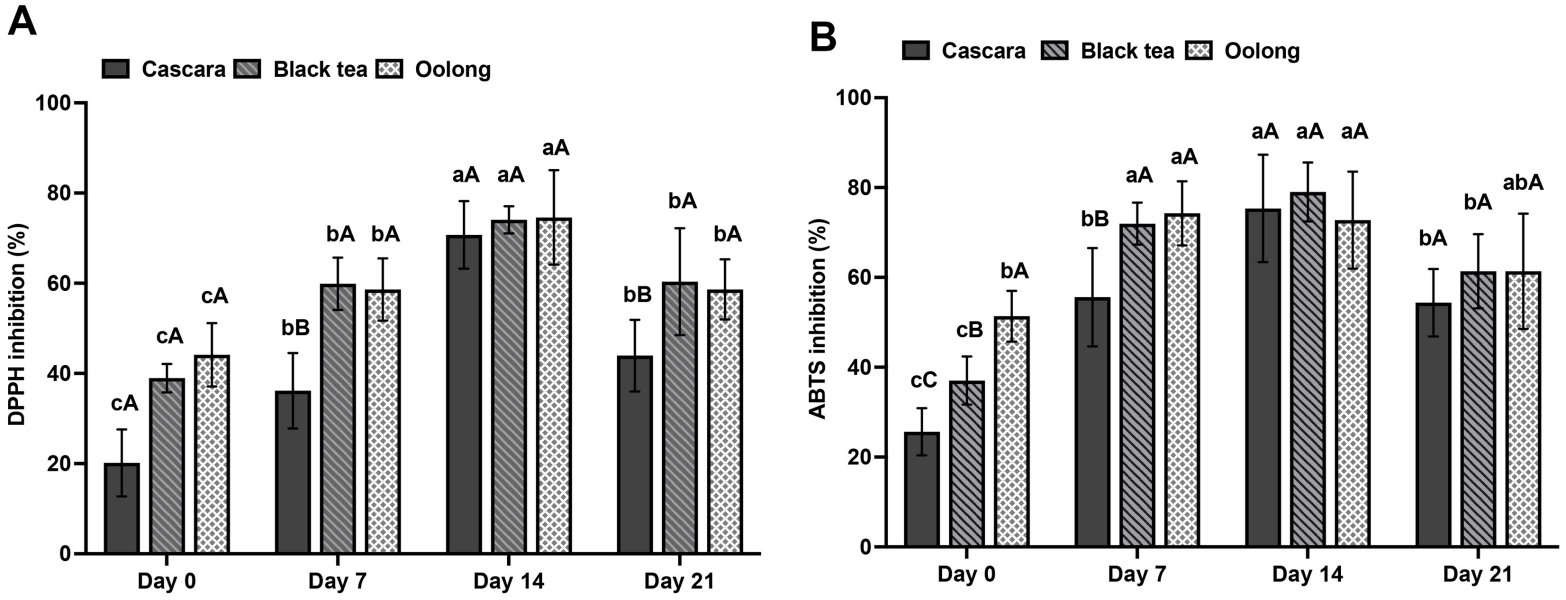
Antioxidant activities of kombucha are expressed by DPPH free radical scavenging (**A**) and ABTS free radical scavenging (**B**). Data are presented as the means of triplicate analysis ± standard deviation. Lowercase letters (a-c) indicate significant differences in antioxidant activities for each kind of kombucha (corresponding to columns of the same color) according to fermentation time interval (*p* < 0.05). Superscript letters (**A-C**) indicate significant differences in antioxidant activities between kombucha derived from cascara, black tea, and oolong tea, respectively, at each fermentation time point (*p* < 0.05).

The antioxidant capacity of cascara kombucha was analyzed by DPPH and ABTS radical scavenging assays (Fig. 3A and 3B). DPPH and ABTS antioxidant activities showed maximal values of 70.7% and 75.3% at 14 days, respectively, before declining again by 21 days. Furthermore, it was observed that the antioxidant potentials of kombucha derived from cascara were similar to those of conventional black tea and oolong tea substrates following a fermentation period of 14 days.

### Analysis of Antibacterial Activities of Cascara Kombucha

The antibacterial efficacy of cascara kombucha was assessed using an agar diffusion assay. The non-fermented cascara substrates did not show any inhibitory effect on the growth of pathogenic bacteria, as shown in Table 1. In contrast, the antibacterial effects of 14-day fermented cascara kombucha were found to be higher than oolong tea and black tea kombucha (Table 1). Furthermore, the kombucha had a selective effect, which inhibits the growth of pathogenic strains such as *E. coli*, *S. aureus*, *S. enterica*, and *V. cholerae*, without affecting the growth of probiotic (Table 1).

### Analysis of Prebiotics Activity

**Fig. 4:**
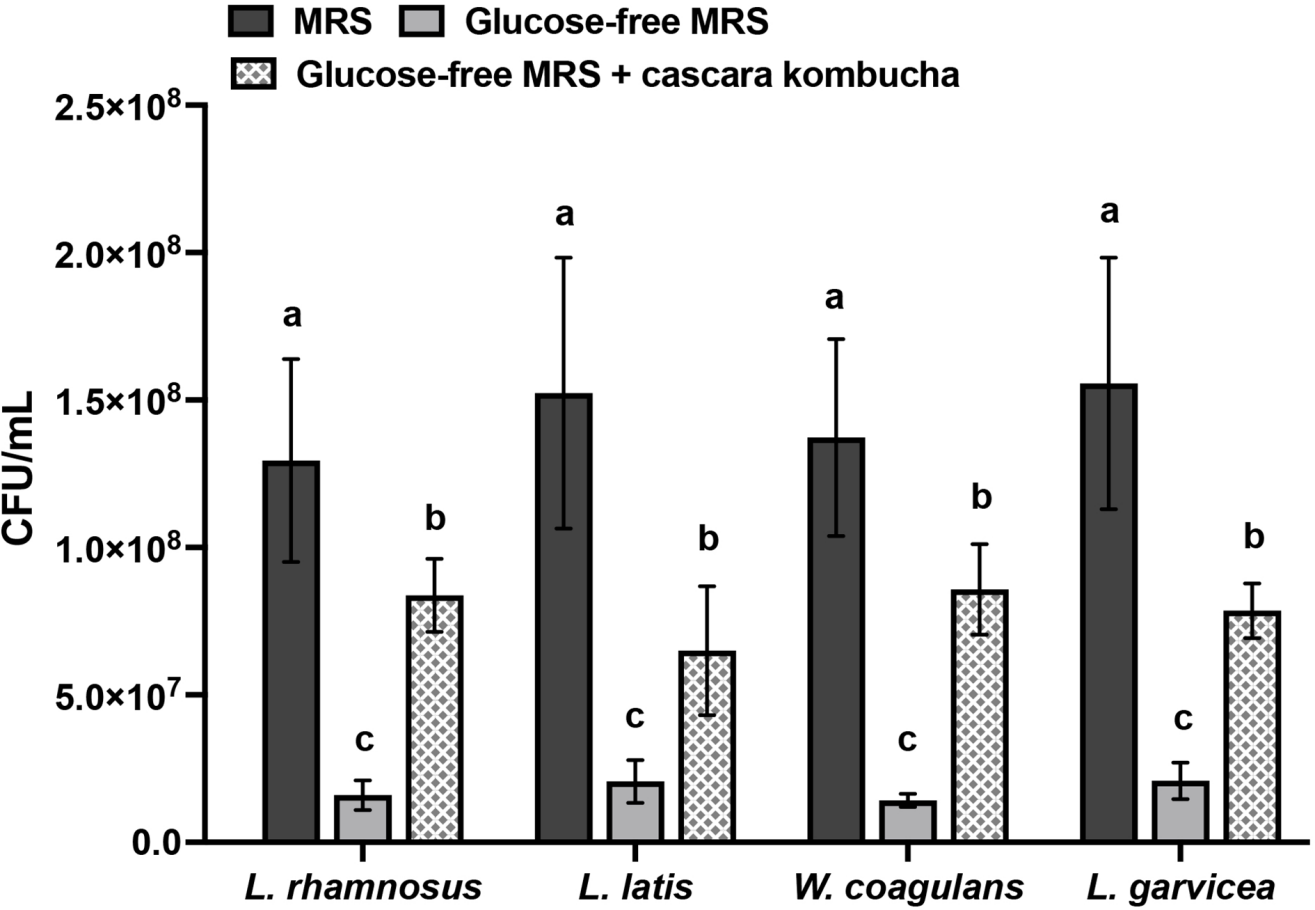
Prebiotic potential of cascara kombucha on promoting the growth of probiotics. Data are presented as the means of triplicate analysis ± standard deviation. Lowercase letters (a-c) indicate significant differences in the growth of each probiotic strain among MRS, free-glucose MRS, and free-glucose MRS with cascara kombucha (*p* < 0.05).

The prebiotic activity of cascara kombucha was assessed by culturing probiotic isolates including *L. gravicae*, *L. latis*, *L. rhamnosus* and *W. coagulans* in a glucose-free MRS medium with or without the addition of 20% cascara kombucha extract. Interestingly, when cultivated in the glucose-free medium alone, no observable growth of these strains was noted (Fig. 4). However, the introduction of 20% cascara kombucha extract into the medium resulted in robust proliferation of all strains, ultimately reaching final cell densities ranging from 6.51 × 10^7^ to 8.37 × 10^7^ CFU/ml (Fig. 4).

### Sensory Evaluation of Cascara Kombucha

**Fig. 5:**
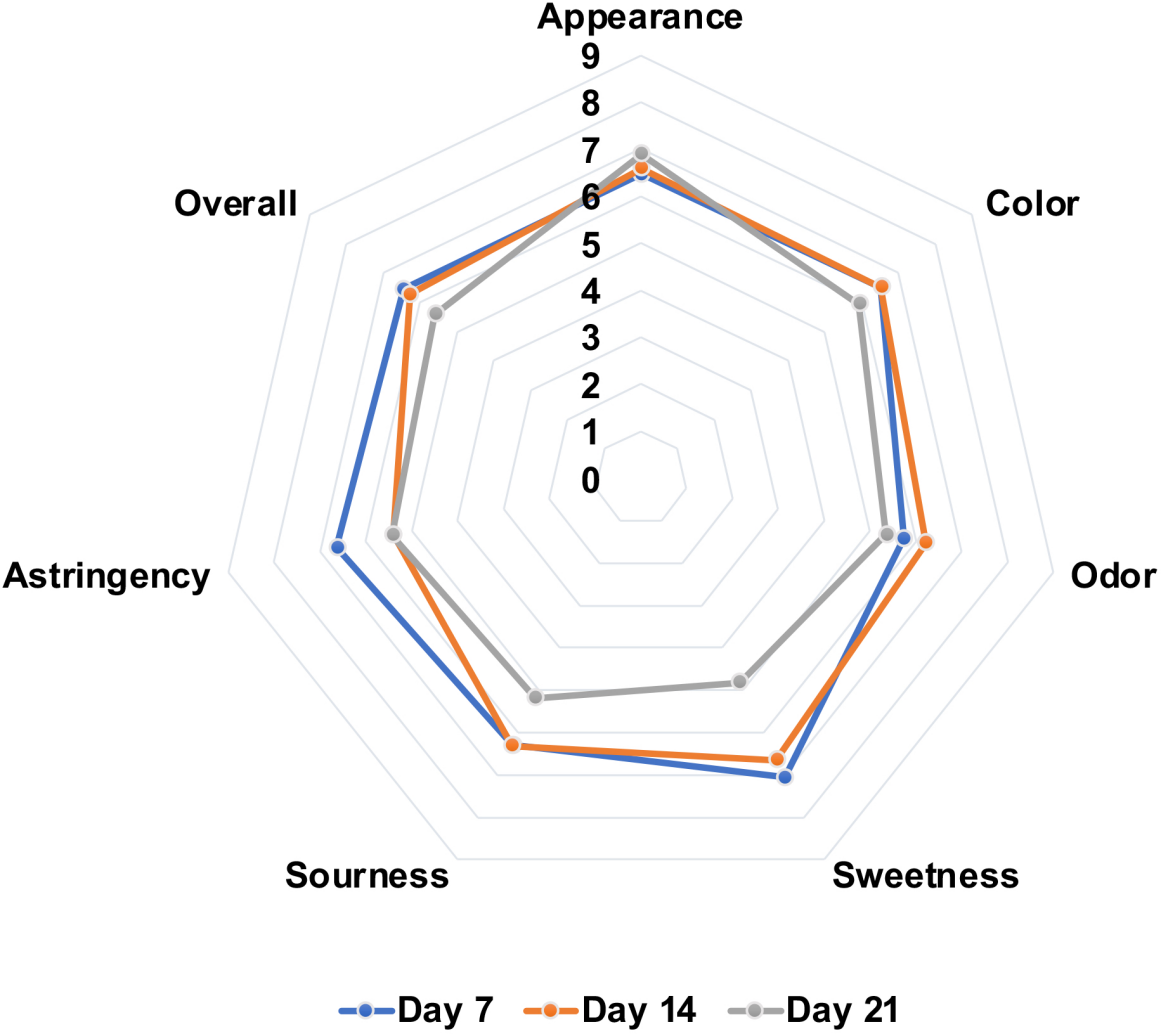
Sensory evaluation of cascara kombucha batches after 7, 14, and 21 days of fermentation.

Sensory analysis revealed temporal differences in sweetness, sourness, and aroma over fermentation time. Extended bioconversion resulted in increased acetic acid and lowered sweetness. Samples fermented for 7 and 14 days were perceived as optimal in terms of balanced sweetness and sourness compared to the strongly acidic 21-day kombucha (*p* < 0.05) (Fig. 5). Furthermore, the 14-day cascara kombucha exhibited the most agreeable aroma (*p* < 0.05) (Fig. 5).

## Discussion

Modulating kombucha fermentation parameters and substituting alternate substrates offers avenues to enhance the sensory properties and nutritional value of the resultant beverage. Various substances such as tea leaves, herbal infusions, fruit juices, vegetables, and seaweed have been subject to inquiry as potential substrates for kombucha. These substrates have been found to impart a wide range of tastes and phytochemicals, as evidenced by previous studies [11, 23, 24]. Cascara, the dried coffee cherry husk, is rich in antioxidants and represents an intriguing candidate ingredient [4].

The advancement of Kombucha fermentation is affected by various factors, including temperature, pH, oxygenation, substrate composition, sugar levels, and fermentation time [25]. Sucrose serves as the principal carbon and energy substrate for facilitating microbial growth and metabolic processes [5]. Microbial development can be hindered by the presence of very high or low sucrose levels, which might induce osmotic stress or carbon limitation, respectively [26]. A recommended sucrose supplementation level of 100 g/l was determined for cascara kombucha, aligning with previous findings suggested that approximately 10% sucrose is appropriate for kombucha production [11].

The decrease in pH and increase in organic acid concentration were also seen during fermentation might be attributed to the microbial activity of the SCOBY on the cascara and sucrose substrates [5]. The initial pH of cascara extract was measured to be 4.8, which gradually decreased to 2.69 and 2.32 after 7 and 14 days, respectively. The reduction in pH indicated the accumulation of acetic acid. The low pH of fermented food has been demonstrated to inhibit the growth of pathogenic bacteria. Moreover, it is advisable for kombucha beverages to keep a pH level within the range of 2.5 to 4.2 [27, 28]. Nevertheless, excessive acidification might have negative effects on the gastrointestinal system [29, 30]. Hence, a fermentation period of 14 days proved to be the optimal duration for achieving the desired attributes of cascara kombucha that align with the prescribed standards for kombucha production.

The presence of polyphenols and flavonoids in cascara contributes to the antioxidant capacity of kombucha. The results of this study indicate a notable increase in the concentrations of polyphenols and flavonoids after a 14-day period, which aligns with prior research that has demonstrated improved extraction of phytochemicals throughout the fermentation process [31]. The process of kombucha bioconversion has the ability to produce many antioxidant metabolites, including catechins and flavonoids [10]. The results of our study demonstrated that cascara kombucha can serve as a significant source of dietary flavonoids, with notable capabilities for scavenging free radicals. Flavonoids have been found to facilitate the process of vascular regeneration, have anti-inflammatory properties, and regulate immune responses [14]. Consequently, cascara kombucha presents a rejuvenating beverage that may possess advantageous health properties ascribed to its phytochemical composition.

The antibacterial efficacy of kombucha is ascribed to its acidic nature throughout the process of fermentation. The presence of microorganisms in the SCOBY leads to the metabolism of sucrose and the subsequent production of several organic acids, including acetic acid, citric acid, and gluconic acid, resulting in a pH range of approximately 2.5 to 3.0 [11]. Previous investigation has provided evidence of the inhibitory properties of kombucha on enteropathogenic bacteria [8]. Our present investigation offers empirical support for the distinct antibacterial capabilities of cascara kombucha against pathogenic bacteria, while concurrently maintaining the advantageous probiotic strains. Cascara kombucha exhibits the potential to serve as a prebiotic agent, hence potentially promoting the proliferation of probiotic microorganisms and contributing to the establishment of a harmonious gut microbiota. The ingestion of kombucha has been shown to promote the proliferation of advantageous bacterial species such as *Proteobacteria* spp., *Bacteroides* spp., and *Lactobacillus* spp., while concurrently diminishing detrimental taxa [32] thus facilitating the equilibrium of the gastrointestinal microbiota.

The determination of the optimal fermentation duration is of utmost importance in the production of cascara kombucha of superior quality. The findings of our study suggest that a fermentation period ranging from 7 to 14 days is the most effective in attaining the necessary sensory characteristics, which include a harmonious combination of sweetness, sourness, and scent. Limiting the duration of fermentation to a period of only 7 days results in a decrease in antioxidant activity, as well as reduced levels of polyphenols and flavonoids, in comparison to a fermentation period of 14 days. It is recommended to prolong the fermentation duration to 14 days in order to optimize the overall quality and potential health advantages provided by the phytochemical elements found in cascara kombucha.

## Conclusion

In conclusion, cascara demonstrates notable potential as a substrate for the production of kombucha beverages that effectively blend desirable sensory characteristics with possible health benefits. The fermenting process greatly increases the concentration of polyphenols and flavonoids, resulting in heightened biological functions, including robust antioxidant and antibacterial activities. These characteristics are in accordance with traditional kombucha made from black tea and oolong tea. In addition, cascara kombucha exhibits a composition that is abundant in prebiotics, which facilitates the proliferation of probiotics. Based on sensory evaluations, it has been observed that cascara kombucha achieves a desirable balance of sweetness and acidity, along with a pleasurable flavor profile, following a fermentation time of 14 days. This makes it a desirable option for individuals who prioritize their health. In addition to its appealing taste, the incorporation of cascara as a medium for the production of kombucha not only results in a unique beverage with various health benefits but also repurposes residual materials from the coffee industry. By engaging in such actions, it actively contributes to the mitigation of environmental damage and advocates for the advancement of sustainable agriculture practices. Although preliminary in vitro evaluation indicated potential health benefits of cascara kombucha. Further investigations employing animal models are essential to comprehensively understand the health advantages of cascara kombucha, providing scientific evidence for product development in the future.

## Figures and Tables

**Table 1:** Antibacterial activities of kombucha beverages, using agar diffusion assay. The inhibition zone calculated in diameter around the well (mm ± SD).

			Inhibition zone diameter (mm)		
			Cascara	Black tea	Oolong
Day 0	Pathogenic bacteria	*E.coli*	-	-	-
		*S. enterica*	-	-	-
		*V. cholerae*	-	-	-
		*S. aureus*	-	-	-
	Probiotics	*L. rhamnosus*	-	-	-
		*L. latis*	-	-	-
		*W. coagulans*	-	-	-
		*L. garvicea*	-	-	-
Day 14	Pathogenic bacteria	*E.coli*	13.3 ± 1.6 ^a^	14.3 ± 1.0 ^a^	9.6 ± 1.3 ^b^
		*S. enterica*	11.5 ± 1.5 ^ns^	11.3 ± 1.8 ^ns^	11.3 ± 1.6 ^ns^
		*V. cholerae*	17.0 ± 2.7 ^a^	17.3 ± 1.9 ^a^	13.7 ± 2.3 ^b^
		*S. aureus*	20.7 ± 1.2 ^a^	21.7 ± 2.4 ^a^	17.0 ± 2.4 ^b^
	Probiotics	*L. rhamnosus*	-	-	-
		*L. latis*	-	-	-
		*W. coagulans*	-	-	-
		*L. garvicea*	-	-	-

Lowercase letters (a - c) indicate significant differences within the line in antibacterial activities between kombucha derived from cascara, black tea, and oolong tea (*p* < 0.05).
